# Psychometric properties of the Brazilian version of the Big Five Inventory

**DOI:** 10.47626/2237-6089-2021-0458

**Published:** 2023-06-27

**Authors:** Paulo Roberto Soares Roiz, Dartiu Xavier da Silveira, Paulo César Ribeiro Barbosa, Murilo Almeida dos Santos Torres, Eliseu da Cruz Moreira, Kelsy Catherina Nema Areco, Ruama Thame Alves de Oliveira, Allan Gama Tazitu, João Ariel Bonar Fernandes, Marcos Gimenes Fernandes, Silvana Kertzer Kasinski

**Affiliations:** 1 Programa de Pós-Graduação em Ciências da Saúde Departamento de Ciências da Saúde Universidade Estadual de Santa Cruz Ilhéus BA Brazil Programa de Pós-Graduação em Ciências da Saúde (PPGCS), Departamento de Ciências da Saúde, Universidade Estadual de Santa Cruz (UESC), Ilhéus, BA, Brazil.; 2 Programa de Orientação e Atendimento a Dependentes Departamento de Psiquiatria Universidade Federal de São Paulo São Paulo SP Brazil Programa de Orientação e Atendimento a Dependentes, Departamento de Psiquiatria, Universidade Federal de São Paulo (UNIFESP), São Paulo, SP, Brazil.; 3 Departamento de Filosofia e Ciências Humanas UESC Ilhéus BA Brazil Departamento de Filosofia e Ciências Humanas, UESC, Ilhéus, BA, Brazil.; 4 Departamento de Ciências Humanas e Filosofia Universidade Estadual de Feira de Santana Feira de Santana BA Brazil Departamento de Ciências Humanas e Filosofia, Universidade Estadual de Feira de Santana (UEFS), Feira de Santana, BA, Brazil.; 5 Faculdade de Ciência da Computação UESC Ilhéus BA Brazil Faculdade de Ciência da Computação, UESC, Ilhéus, BA, Brazil.; 6 Faculdade de Medicina UESC Ilhéus BA Brazil Faculdade de Medicina, UESC, Ilhéus, BA, Brazil.; 7 Departamento de Informática em Saúde Escola Paulista de Medicina UNIFESP São Paulo SP Brazil Departamento de Informática em Saúde, Escola Paulista de Medicina, UNIFESP, São Paulo, SP, Brazil.; 8 Departamento de Psiquiatria e Psicologia Médica UNIFESP São Paulo SP Brazil Departamento de Psiquiatria e Psicologia Médica, UNIFESP, São Paulo, SP, Brazil.; 9 Faculdade de Educação Física UESC Ilhéus BA Brazil Faculdade de Educação Física, UESC, Ilhéus, BA, Brazil.; 10 Departamento de Ortopedia e Traumatologia UNIFESP São Paulo SP Brazil Departamento de Ortopedia e Traumatologia, UNIFESP, São Paulo, SP, Brazil.

**Keywords:** Personality, BFI, cross-cultural adaptation

## Abstract

**Introduction:**

There is growing interest in the fields of psychiatry and psychology in investigating the relationship between personality and psychopathology. The Big-5 is a model developed to investigate five personality dimensions: Extroversion, Agreeableness, Conscientiousness, Neuroticism, and Openness. In the present study, we describe the process of translation into Brazilian Portuguese and adaptation of a free tool to evaluate the Big-5 model: The Big-5 Inventory (BFI). The instrument has 44 items with a Likert response scale ranging from 1 to 5.

**Objectives:**

To translate and adapt the BFI into Brazilian Portuguese.

**Methods:**

The adaptation was conducted in the following steps: 1) Translation, 2) Evaluation Committee, 3) Back-translation, 4) Pilot study, 5) Evaluation Committee, and 6) Application. The sample comprised 490 participants from various regions of Brazil. The participants’ ages ranged from 18 to 71 years, most of them had completed high school (62.9%), and the majority were women (75%).

**Results:**

A model with the following fit indexes was found: χ^2^/df: 1.954; goodness fit index (GFI): 0.924; comparative fit index (CFI): 0.920; and root mean square error of approximation (RMSEA): 0.044.

**Conclusion:**

The results are suggestive that the Brazilian version of this instrument has good psychometric properties and represent a cost-free option for investigating associations with the Big-5 in psychiatry.

## Introduction

One of the best-known constructs in the study of personality is the Big Five Factors theory (Big-5), which involves five dimensions of personality: Neuroticism, Conscientiousness, Agreeableness, Openness, and Extraversion.^1^ Each trait encompasses different characteristics of an individual: Openness is related to creativity and imagination; Conscientiousness is related to organization and reliability; Extroversion is a tendency towards sociability and assertiveness; Agreeableness is a tendency towards prosocial attitudes and altruism; and Neuroticism is a tendency to sadness and negative emotions.^2^

Researchers have been investigating the association of the Big Five model with psychiatric disorders, psychological well-being and general well-being.^3 - 6^ A recent systematic review of stroke patients showed that personality may indicate prognosis for treatment. The study demonstrated that post-stroke patients who had high Neuroticism scores were more likely to be affected by depression.^7^ Another study identified a strong association between one’s ability to delay gratification and the Conscientiousness trait, which is especially relevant to health problems such as overweight, drug abuse and risky sexual behavior.^8^

In Brazil, the most used inventory for evaluating the Big-5 model is the NEO Personality Inventory (NEO-PI-R), prepared by Costa and McRae,^9^ adapted to Brazilian Portuguese by Carmen Flores Medonza, and published by Vetor.^10^ Its use in research is limited, however, because it is restricted to psychologists, and use is prohibited for other professionals whose research could greatly benefit from assessment of the Big-5 model, such as psychiatrists, neurologists, and neuroscientists. Furthermore, the NEO-PI-R can only be acquired by purchasing it for a fee, which poses an additional difficulty, especially at a time when the country’s research funding is suffering from drastic budget cuts.

The Big-5 Inventory (BFI) is an instrument for use in research, consisting of 44 Likert-type questions, designed to assess the five major personality factors. The BFI is an instrument that has been used in research in several countries,^2^ for example: in France with higher education students with an average age of 21 years^11^ ; in the Bolivian Amazon area with farmers aged from 20 to 88 years^12^ ; in Rwanda and the Philippines, with young people and adolescents^13^ with average ages of 21 and 15.5 years for each country, respectively; with young Chinese people (average age of 25.3 years) residing in Germany^14^ ; in England with adults aged from 20 to 80 years^15^ ; and in New Zealand with construction workers aged from 20 to 50 years.^16^

Based on the evidence presented above, it is necessary to make a free instrument available to assess the five major personality traits, which would be very useful for researchers in Brazil as well.^17^

## Methods

The present study consisted of the translation and cross-cultural adaptation of the original instrument from English (North American) into Brazilian Portuguese. The instrument is free to use for research purposes.

The translation process was based on the general guidelines described by Hungerbuhler and Wang^17^ and the International Test Commission,^18^ and consisted of the following steps:

1. Translation: two independent translators translated the original Big Five Inventory into Brazilian Portuguese.
2. Evaluation Committee: two translators with experience in Psychology and Psychiatry analyzed and produced a synthesis version.
3. Back-translation: a bilingual American researcher (English and Portuguese) performed a back-translation of the synthesis version.
4. Pilot study: the version was administered using Google forms to 46 participants in a test and re-test format.
5. Evaluation Committee: a group of neuropsychology researchers conducted a new analysis, making some alterations, and producing a definitive version. The evaluation committee was composed of two Ph.D. professors with extensive experience in psychiatry and psychology; three Master’s students in health sciences, one psychologist, and two undergraduate students. The items were presented on a slide, and participants were asked which items they agreed with and which items they would suggest changing.
6. Final administration: the definitive version of the instrument was administered and validated with confirmatory factor analysis (CFA).

### Sample

The sample size was based on 10:1 ratio,^17^ to achieve a proportion of 10 subjects for each item of the instrument. This ratio is widely used for instrument validation.

Sample selection can be characterized as non-probabilistic and by convenience, recruiting 490 participants (369 women, 118 men, and three non-binary people) (M = 35.01, standard deviation [SD]: 11.99), with different educational levels. Majorities had completed college (306), were single (297), and were from the northeast region of Brazil (327).

### Procedures

Administration of the definitive version of the Brazilian Portuguese Big Five Inventory instrument was conducted between May 3, 2021, and June 13, 2021, via Google Forms. A link was posted on social networks along with an ad that invited several people to answer the survey. The ad made it clear that the survey should only be answered by people over 18. The ad also stated how long it should take to complete the instrument. After clicking on the survey link, the participant would first be asked to choose whether or not to sign the informed consent form, which contained all the information about the project, as well as contact information. The present study was approved by the Research Ethics Committee (protocol 36899520.6.0000.5526) at the Universidade Estadual de Santa Cruz (UESC), in accordance with Conselho Nacional de Saúde/Brazilian Ministry of Health (CNS/MS) Resolution MS n. 466/2012.

### Data analysis

AMOS 23.0 was used to test the models. The maximum likelihood model (MLE) was used, respecting a minimum of 10 observations per item.^19^ After specifying and estimating the models, their applicability was evaluated against a set of fit indices. The fit indices analyzed were chi-square by degrees of freedom (χ^2^ /df), for which values greater than 2 are acceptable^20^ ; the comparative fit index (CFI) and goodness of fit index (GFI), which can both vary from 0 to 1, where values greater than 0.90 indicate an adequate model according to Bentler and Bonnet^20^ ; the root mean square error of approximation (RMSEA), for which a value less than 0.06 indicates acceptable adequacy^21^ ; and the Akaike information criterion (AIC), which evaluates the simplicity of the model by testing the lowest value in the model.

Composite reliability (CR) and average variance extracted (AVE) were also analyzed, both of which enable us to assess the quality of the instrument.^22^ Acceptable reference values for CR and AVE are greater than 0.7^22^ and greater than or equal to 0.5,^23^ respectively. Cronbach’s alpha was also calculated and we set the reference value at > 0.7.

## Results

The original, translated, and back-translated versions of the Big Five Inventory are shown in Table 1 . The review committee decided to retain the same instrument title, adding the language version to it, as follows: The Big Five Inventory, Brazilian Portuguese version (Supplementary Material S1, available online-only).

**Table 1:** Original, translated, and back-translated versions of the Big Five Inventory

Big Five Inventory (Big-5)	Synthesis version	Back-translation	Definitive version

	*Eu me considero uma pessoa que...*	I consider myself to be a person that...	*Eu me considero uma pessoa que...*
1. Is talkative.*	*Gosta de conversar. É comunicativa.*	Likes to talk/have conversations. Is communicative (is a good communicator).*	*Gosta de conversar é comunicativa.*
2. Tends to find fault with others.†	*Tende a ser crítica com os outros.*	Tends to be critical of others.^†^	*Tende a criticar os outros.*
3. Does a thorough job.	*É minuciosa e detalhista no trabalho.*	Is thorough and detailed/pays attention to detail at work.^†^	*É minuciosa e detalhista no trabalho.*
4. Is depressed, blue.	*Depressiva, triste.*	Depressed, sad.^†^	*Depressiva, triste.*
5. Is original, comes up with new ideas.	*É original, tem ideias novas.*	Is original, has new ideas.^†^	*É original, tem ideias novas.*
6. Is reserved.	*É reservada.*	Is reserved.*	*É reservada.*
7. Is helpful and unselfish with others.	*É prestativa e solidária com os outros.*	Is helpful and supportive of others.^†^	*É generosa e não é egoísta com outras pessoas.*
8. Can be somewhat careless.	*Pode ser um pouco descuidada nas tarefas*	Can be a little careless with tasks.^†^	*Pode ser desleixada para fazer as coisas.*
9. Is relaxed, handles stress well.	*É tranquila, lida bem com estresse.*	Is calm/easygoing, deals well with stress.^†^	*É tranquila, lida bem com estresse.*
10. Is curious about many different things.	*É curiosa, interessada em várias coisas diferentes.*	Is curious, interested in many different things.^†^	*Se interessa por áreas diferentes do conhecimento.*
11. Is full of energy.	*É cheia de energia.*	Is full of energy.*	*É cheia de energia.*
12. Starts quarrels with others.	*Começa discussões com os outros.*	Argues with others.^†^	*Inicia bate-boca com outros.*
13. Is a reliable worker.	*É confiável no trabalho.*	Is reliable/trustworthy at work.^†^	*É confiável no trabalho.*
14. Can be tense.	*Pode ser tensa.*	Can be stressed.^†^	*Pode ser tensa.*
15. Is ingenious, a deep thinker.	*Que pensa profundamente nas coisas.*	That thinks deeply about things.^†^	*É inovadora, pensa profundamente nas coisas.*
16. Generates a lot of enthusiasm.	*Gera muito entusiasmo.*	Creates lots of enthusiasm.*	*Gera muito entusiasmo.*
17. Has a forgiving nature.	*Desculpa, perdoa os outros.*	Excuses, forgives others.^†^	*Desculpa, perdoa os outros.*
18. Tends to be disorganized.	*Tende a ser desorganizada.*	Tends to be disorganized.^†^	*Tende a ser desorganizada.*
19. Worries a lot.	*Se preocupa muito.*	Worries often/a lot.^†^	*Se preocupa muito, em excesso.*
20. Has an active imagination.	*Tem uma imaginação fértil.*	Has a creative/good imagination.^†^	*Tem uma imaginação fértil.*
21. Tends to be quiet.	*Tende a ser quieta.*	Tends to be quiet.*	*Tende a ser quieta.*
22. Is generally trusting.	*Geralmente confia, acredita nos outros.*	Generally trusts, believes in others.^†^	*Geralmente confia, acredita nos outros.*
23. Tends to be lazy.	*Tende a ser preguiçosa,*	Tends to be lazy.*	*Tende a ser preguiçosa.*
24. Is emotionally stable, not easily upset.	*É emocionalmente estável, não se perturba facilmente.*	Is emotionally stable, is not easily disturbed.^†^	*É emocionalmente estável, não se perturba facilmente.*
25. Is inventive.	*É inventiva.*	Is creative.^†^	*É inventiva.*
26. Has an assertive personality.	*É assertiva, não tem medo de expressar o que sente.*	Is assertive, is not afraid to express what i feel/my feeling.^†^	*É assertiva, não tem medo de expressar o que sente.*
27. Can be cold and aloof.	*Pode ser fria e indiferente com os outros.*	May be cold and indifferent towards others.^†^	*Às vezes é indiferente com os outros.*
28. Perseveres until the task is finished.	*Persevera até concluir as tarefas,*	Completes tasks/sees tasks through to the end.^†^	*Persevera até concluir as tarefas.*
29. Can be moody.	*Fica triste ou irritada facilmente.*	Easily gets sad or irritated.^†^	*É temperamental e instável emocionalmente.*
30. Values artistic, aesthetic experience.	*Valoriza experiências artísticas e estéticas.*	Values artistic and expressive experiences.^†^	*Valoriza experiências artísticas e estéticas.*
31. Is sometimes shy, inhibited.	*Às vezes é tímida, inibida.*	Is sometimes timid, shy.^†^	*Às vezes é tímida, inibida.*
32. Is considerate and kind to almost everyone.	*É gentil e tem consideração com quase todo mundo.*	Is kind and considerate of almost everyone.^†^	*É boa e atenciosa com quase todo mundo.*
33. Does things efficiently.	*Faz as coisas com eficiência.*	Does things efficiently.*	*Faz as coisas com eficiência.*
34. Remains calm in tense situations.	*Se mantem calma em situações tensas.*	Stays/remains calm in tense/difficult situations.^†^	*Se mantém calma em situações tensas.*
35. Prefers work that is routine.	*Prefere trabalhos com rotina.*	Prefers work with a routine.^†^	*Gosta de rotina.*
36. Is outgoing, sociable.	*É extrovertida e sociável.*	Is extroverted and sociable.^†^	*É extrovertida e sociável.*
37. Is sometimes rude to others.	*Às vezes é rude, mal educada, com os outros.*	Is sometimes rude, unpolite, with others.^†^	*Às vezes é grosseira com outras pessoas.*
38. Makes plans and follows through with them.	*Faz os planos e não se desvia deles.*	Makes plans and does not change them.^†^	*Cumpre, finaliza os planos que faz.*
39. Gets nervous easily.	*Fica nervosa facilmente.*	Becomes/ is made nervous easily.^†^	*Fica nervosa facilmente.*
40. Likes to reflect, play with ideas.	*Gosta de refletir, brincar com as ideias.*	Likes to reflect, play with ideas.^†^	*Gosta de refletir, jogar com as ideias.*
41. Has few artistic interests.	*Não tem muitos interesses em arte.*	Does not have much interest in art.^†^	*Tem poucos interesses artísticos.*
42. Likes to cooperate with others.	*Gosta de cooperar com outros.*	Likes to cooperate/work with others.^†^	*Gosta de cooperar com outros.*
43. Is easily distracted.	*Se distrai facilmente.*	Is easily distracted.*	*Se distrai facilmente.*
44. Is sophisticated in art, music, or literature.	*É sofisticada em arte, música ou literatura.*	Is sophisticated/refined in art, music or literature.*	*É sofisticada em arte, música ou literatura.*

* There were no changes between the original version and the back translation.

^†^ Changes were made between the original version and the back translation or from the adjusted synthesis version to the definitive version.

The mean item response values ranged from 1.48 ± 0.73 (item 20) to 3.31 ± 0.77 (item 15). The univariate normality values tended to lie in a range associated with a normal distribution. The mean and standard deviations of the factors were as follows: Extroversion (M = 3.30; SD = 0.74), Agreeableness (M = 3.68; SD = 0.54), Conscientiousness (M = 3.74; SD = 0.64), Neuroticism (M = 2.95; SD = 0.81), and Openness (M = 3.78; SD = 0.59) ( Table 2 ).

**Table 2:** Participant data

Variable	n (%)
Gender	
Female	369 (75.0)
Male	118 (24.0)
Non-binary	3 (1.0)
Age (years)	
18 to 30	221 (45.1)
31 to 40	125 (26.9)
41 to 50	62 (15.3)
51 to 60	42 (7.5)
61 to 71	20 (4.5)
Education level	
Incomplete primary school	3 (0.6)
Complete primary school	4 (0.8)
Incomplete secondary school	3 (0.6)
Complete secondary school	51 (10.4)
Incomplete higher education	122 (24.9)
Complete higher education	306 (62.9)
Marital status	
Married	162 (33.1)
Divorced	27 (5.5)
Single	297 (60.6)
Widowed	4 (0.6)
Geographic region	
Northeast	327 (66.7)
Midwest	13 (2.7)
South	57 (11.6)
Southeast	60 (12.2)
North	33 (6.7)

Table 3 shows the result of the confirmatory factor analysis.

**Table 3:** CFA fit indices for the different models tested.

	χ^2^/DF	GFI	CFI	RMSEA	AIC
Model 1	5.619	0.609	0.423	0.097	4100.62
Model 2	3.195	0.788	0.730	0.067	2386.94
Model 3	3.179	0.763	0.683	0.067	3031.75
Model 4	3.920	0.765	0.689	0.077	2201.794
Model 5	3.334	0.805	0.756	0.069	1879.762
Model 6	5.391	0.675	0.533	0.095	2973.713
Model 7	1.961	0.922	0.912	0.044	639.704
Model 8	1.954	0.924	0.920	0.044	637.988
Model 9	9.911	0.898	0.837	0.135	230.227
Model 10	5.178	0.937	0.784	0.092	175.802
Model 11	8.674	0.890	0.779	0.125	270.199
Model 12	4.289	0.958	0.938	0.082	117.789
Model 13	4.468	0.932	0.867	0.084	196.370

AIC = Akaike information criterion; CFA = confirmatory factor analysis; CFI = comparative fit index; GFI = goodness of fit index; RMSEA = root mean square error of approximation; χ^2^/DF = chi-square by degrees of freedom.

Model 1 – Unifactorial; Model 2 – Removed items A: 2r, 37r, 12r, 22, Ab: 35r; Model 3 – All items; Model 4 – Removed items 2, 17, 22, 27, 37, 4, 19, 35. No correlation between factors – the Benet-Martinez and John model ^24^ ; Model 5 – Removed items 2, 17, 22, 27, 37, 4, 19, 35, with correlation between factors – Chiorri et al. ^25^ ; Model 6 – Five primary factors and two second-order factors ^26^ ; Model 7 – Removed items E: 6, 11, 21, 31, 36, A: 2, 12, 27, 37, C: 3, 13, 33, 38, N: 4, 29, Ab: 20, 30, 35, 41 added covariances between item errors: 14-19, 40-44; Model 8 – Removed items E: 6, 21, 31, 36, A: 2, 12, 22, 27, 37, C: 3, 13, 33, 38, N: 4, 29, Ab: 20, 30, 35, 41, added covariances between item errors: 11-23, 8-18, 9-10, 40-44; Model 9 – Extroversion factor items only; Model 10 – Agreeableness factor items only; Model 11 – Conscientiousness factor items only; Model 12 – Neuroticism factor items only; Model 13 – Openness factor items only.

Seven models were tested, three of which have been used in prior literature (M4, M5, and M6). The M4 and M5 models removed items 2, 4, 17, 19, 22, 27, 35, and 37, the former without correlation between the factors and the latter correlating the factors. Model M6 was estimated without the aforementioned items, but with inclusion of two second-order factors. In M1, a single factor was used containing all items. In M3, all items that had factor loadings less than 0.3 were removed (2, 12, 22, 35, 37). In M7, items that presented residual covariances were removed until the model was adjusted.

The original BFI model with five factors and 44 items (M3) had several adjustment problems (CFI and GFI < 0.90), suggesting that this factor structure does not satisfactorily represent the data. Analysis of the coefficients (standardized and non-standardized) for model 3 (BFI) revealed that item loadings ranged from 0.05 (item 12) to 0.79 (item 39) and all of these parameters were significant at p < 0.001. The factor correlations ranged from -0.46 (p < 0.001), for Neuroticism and Conscientiousness, to 0.53 (p < 0.001), for Extroversion and Agreeableness.

**Figure 1:**
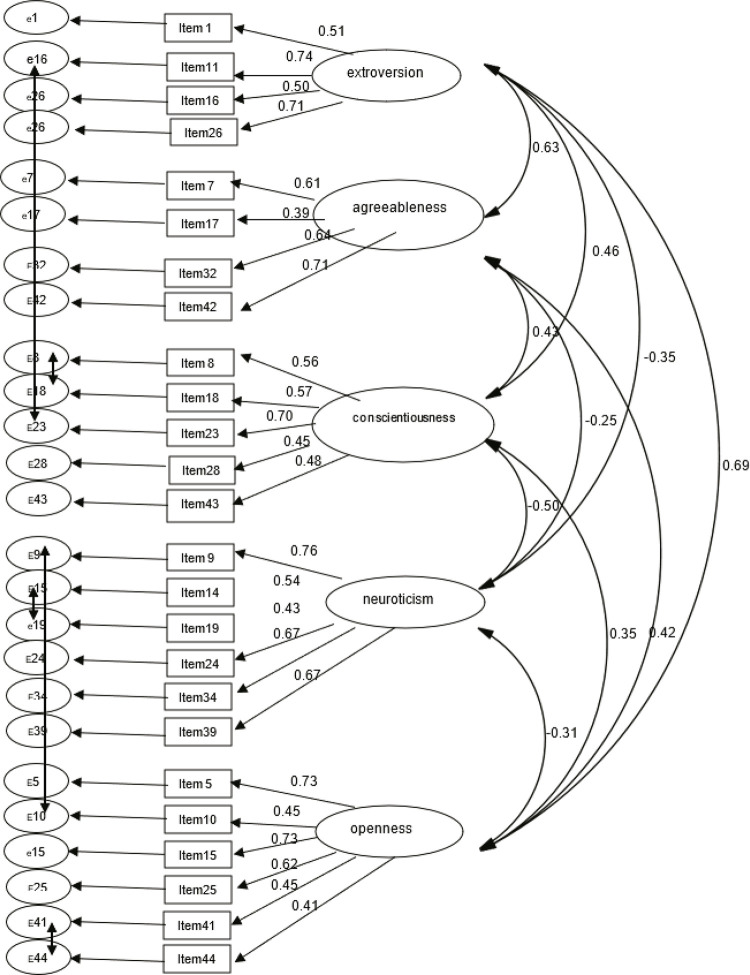
Structural equation modeling - model 8

Analysis of the standardized and non-standardized coefficients of the factor loadings of Model 8 (BFI) revealed that the item loadings ranged from 0.39 (item 17) to 0.76 (item 9) and all of these parameters were significant at p < 0.001 (R2 > 0.19). The correlations between the residual errors of the following items were: items 11 and 23 (0.28), items 8 and 18 (0.23); items 9 and 10 (0.24); items 14 and 19 (0.18); items 9 and 10 (0.24); items 40 and 44 (0.19). The correlations between factors ranged from -0.50 (p < 0.001), for Neuroticism and Conscientiousness, to 0.69 (p < 0.001), for Extroversion and Openness ( Figure 1 ).

Covariances between the following items were added: 11 – *É cheia de energia* ; 23 – *Tende a ser preguiçosa* ; 8 – *Pode ser desleixada para fazer as coisas* ; 23 – *Tende a ser preguiçosa* ; 9 – *É tranquila, lida bem com estresse* ; 10 – *É curiosa, interessada em várias coisas diferentes* ; 40 – *Gosta de refletir, brincar com as ideias* ; 44 – *É sofisticada em arte, música ou literatura* .

The results in Table 4 demonstrate that, in general, all factors presented adequate CR, with values above 0.7. The factors had acceptable AVE values, except for Openness, which was borderline.

**Table 4:** Model 8 CR and AVE

Factors	Items	Λ	ɛ	CR	AVE
Extroversion	2	0.51	0.26		
	11	0.71	0.5	0.79	0.5
	16	0.74	0.55		
	26	0.5	0.25		
Agreeableness	7	0.61	0.38		
	17	0.39	0.15		
	32	0.64	0.41	0.79	0.5
	42	0.71	0.5		
Conscientiousness	8	0.56	0.32		
	18	0.57	0.33		
	23	0.7	0.5	0.83	0.5
	28	0.48	0.23		
	43	0.48	0.23		
Neuroticism	9	0.76	0.58		
	14	0.54	0.25		
	19	0.43	0.16	0.86	0.51
	24	0.67	0.45		
	34	0.65	0.42		
	39	0.67	0.46		
Openness	5	0.73	0.53		
	10	0.45	0.2		
	15	0.73	0.59	0.85	0.49
	25	0.62	0.38		
	40	0.45	0.2		
	44	0.41	0.17		

AVE = average variance extracted; CR = composite reliability; Λ = factor loading.

Cronbach’s alpha reached adequate indices except for the Agreeableness factor: Agreeableness, 0.632; Extroversion, 0.8; Openness, 0.74; Neuroticism, 0.82; and Conscientiousness, 0.76.

## Discussion

The Big Five Inventory, Brazilian Portuguese version, showed, in general, adequate results in terms of reliability. Data are consistent with the Italian, Danish, Dutch, German, and English versions.^2 , 27 - 30^ This demonstrates the instrument’s high degree of applicability.

The process of translation followed the stages of translation, synthesis, back-translation, committee analysis, pre-test and application, as the model is related to that described by Beaton et al.^31^

Care was taken to use shorter sentences in the translation from English to Portuguese, since, according to Pallson et al.,^27^ long phrases hinder the ability of participants in pain and the elderly to use the instrument.

Cronbach’s α values were above the recommended cutoffs (0.7) for the Conscientiousness, Neuroticism, Openness, and Extraversion items, demonstrating good internal consistency. However, the Agreeableness domain had scores below the ideal value, which corroborates findings for the German, Danish, and Italian versions, at 0.67, 0.66, and 0.69 respectively.^27 , 28 , 30^

Regarding the construct validity of the Big Five Inventory, CFA showed that the model (M8) with 25 items had better fit indexes than the original model (M3) with 44 items. The models suggested by Benet-Martinez and John,^25^ Chiorri et al.,^26^ and Jang et al.^27^ had fit indices below the reference values. Model 7 had good fit indices, but the extraversion factor only had three items, while Model 8, with four items for the extraversion factor had better results.

The individual analysis of the parameters estimated showed that the loadings of most items onto their respective factors were greater than 0.40, except for item 17.

Regarding reliability, it is known that CR is a more accurate indicator of precision than Cronbach’s alpha, because CR factor loads are free to vary among themselves, whereas in Cronbach’s alpha, factor loads are fixed as equal. The CR is therefore able to produce better adjusted accuracy indices. All factors had values above 0.7, which indicates homogeneity between items.

Regarding the AVE, all results were above the reference value (0.5), except for the Openness factor, which had a borderline index. This means that most factors (latent variables) explain more than half of the variance of all of the items they contain, according to Valentini and Damásio.^32^

The validated model (M8) retained 25 items and maintained the five factors that support the Big Five theory.^2^ Church and Burke^33^ point out difficulties with use of CFA in personality instruments, since there are restrictions to assessment of the personality structure. The original model (M3), for example, had low fit indices. Other models observed in the literature also failed to achieve adequate fit indices, Benet-Martinez and John,^24^ Danu,^34^ Little et al.,^35^ Marsh et al.^36^

The present study has some limitations, including the following: the sample was selected by convenience and was non-probabilistic; and the sample contained majorities of females and northeast Brazilian participants. Invariance was not a study goal because groups are not balanced by gender or region. We suggest that future studies perform invariance analysis.

## Conclusion

Research associated with personality has increased considerably, which highlights the need for a measurement instrument that can provide more accurate measurements. The Big Five Inventory is used worldwide and proves to be this instrument.

There is also a need for free instruments to support research producing evidence, since this will facilitate replicability and increase researchers’ access to studies involving personality. In Brazil there is a need for more studies involving personality and this instrument could substantially contribute to increase such research.
