# Supplementary material for: Psychometric properties of the Brazilian version of the Big Five Inventory
Source: Trends Psychiatry Psychother. 2023 Jun 27;45:e20210458. doi: 10.47626/2237-6089-2021-0458 (PMC10416253; doi:10.47626/2237-6089-2021-0458)
Supplement: Supplementary file 1 [file 2238-0019-trends-45-e20210458-suppl1.pdf]

Supplementary Material S1

De forma geral, como eu sou. Aqui você verá um número de características que pode ou não se aplicar a você. Por exemplo, você concorda que você é alguém que gosta de passar tempo com outros? Por favor escreva um número próximo à cada declaração que indica a extensão que você concorda ou discorda com a declaração.
1. Discordo totalmente
2. Discordo um pouco
3. Nem concordo, nem discordo
4. Concordo um pouco
5. Concordo totalmente

The Big Five Inventory, Brazilian Portuguese version

Versão definitiva
Eu me considero uma pessoa que...
1. Gosta de conversar, é comunicativa.
2.Tende a criticar os outros.
3.É minuciosa e detalhista no trabalho.
4.Depressiva, triste.
5.É original, tem ideias novas.
6.É reservada.
7.É generosa e não é egoísta com outras pessoas.
8.Pode ser desleixada para fazer as coisas.
9.É tranquila, lida bem com estresse.
10.Se interessa por áreas diferentes de conhecimento.
11.É cheia de energia.
12.Inicia bate-boca com outros.
13.É confiável no trabalho.
14.Pode ser tensa.
15.É inovadora, pensa profundamente nas coisas.
16.Gera muito entusiasmo.
17.Desculpa, perdoa os outros.
18.Tende a ser desorganizada.
19.Se preocupa muito, em excesso.
20.Tem uma imaginação fértil.
21.Tende a ser quieta.
22.Geralmente confia, acredita nos outros.
23.Tende a ser preguiçosa.
24.É emocionalmente estável, não se perturba facilmente.
25.É inventiva.
26.É assertiva, não tem medo de expressar o que sente.
27.Às vezes é indiferente com os outros.
28.Persevera até concluir as tarefas.
29.É temperamental e instável emocionalmente.
30.Valoriza experiências artísticas e estéticas.
31.Às vezes é tímida, inibida.

http://doi.org/10.47626/2237-6089-2021-0458

32.É boa e atenciosa com quase todo mundo.
33.Faz as coisas com eficiência.
34.Se mantem calma em situações tensas.
35.Gosta de rotina.
36.É extrovertida e sociável.
37.Às vezes é grosseira com outras pessoas.
38.Cumpre, finaliza os planos que faz.
39.Fica nervosa facilmente.
40.Gosta de refletir, jogar com as ideias.
41.Tem poucos interesses artísticos.
42.Gosta de cooperar com outros.
43.Se distrai facilmente.
44.É sofisticada em arte, música ou literatura.
